# Neuropsychiatric Symptom Clusters in Stroke and Transient Ischemic Attack by Cognitive Status and Stroke Subtype: Frequency and Relationships with Vascular Lesions, Brain Atrophy and Amyloid

**DOI:** 10.1371/journal.pone.0162846

**Published:** 2016-09-15

**Authors:** Adrian Wong, Alexander Y. L. Lau, Jie Yang, Zhaolu Wang, Wenyan Liu, Bonnie Y. K. Lam, Lisa Au, Lin Shi, Defeng Wang, Winnie C. W. Chu, Yun-yun Xiong, Eugene S. K. Lo, Lorraine S. N. Law, Thomas W. H. Leung, Linda C. W. Lam, Anne Y. Y. Chan, Yannie O. Y. Soo, Eric Y. L. Leung, Lawrence K. S. Wong, Vincent C. T. Mok

**Affiliations:** 1 Department of Medicine and Therapeutics, The Chinese University of Hong Kong, Hong Kong, China; 2 Department of Imaging and Interventional Radiology, The Chinese University of Hong Kong, Hong Kong, China; 3 Department of Psychiatry, Lui Che Woo Institute of Innovative Medicine, The Chinese University of Hong Kong, Hong Kong, China; 4 Therese Pei Fong Chow Research Centre for Prevention of Dementia, The Chinese University of Hong Kong, Hong Kong, China; 5 Institute of Neuroscience and the Second Affiliated Hospital of Guangzhou Medical University and Key Laboratory of Neurogenetics and Channelopathies of Guangdong Province and Ministry of Education of China, Guangzhou, 510260, China; 6 Department of Neurology, Jinling Hospital, Nanjing University School of Medicine, Nanjing, China; 7 Department of Nuclear Medicine, Hong Kong Sanatorium and Hospital, Hong Kong, China; Taipei Veterans General Hospital, TAIWAN

## Abstract

**Background:**

The objectives of this study are 1) to examine the frequencies of neuropsychiatric symptom clusters in patients with stroke or transient ischemic attack (TIA) by cognitive level and stroke subtype; and 2) to evaluate effect of demographic, clinical, and neuroimaging measures of chronic brain changes and amyloid upon neuropsychiatric symptom clusters.

**Methods:**

Hospital-based, cross-sectional study. 518 patients were administered the Neuropsychiatric Inventory (NPI) 3–6 months post index admission. NPI symptoms were classified into four symptom clusters (Behavioral Problems, Psychosis, Mood Disturbance & Euphoria) derived from a confirmatory factor analysis of the 12 NPI items. Multivariable logistic regression was used to determine independent associations between demographic, clinical and neuroimaging measures of chronic brain changes (white matter changes, old infarcts, whole brain atrophy, medial temporal lobe atrophy [MTLA] and frontal lobe atrophy [FLA]) with the presence of NPI symptoms and all symptom clusters except euphoria. ^11^C-Pittsburg Compound B Positron Emission Tomography (^11^C-PiB PET) was performed in 24 patients to measure amyloid retention for Alzheimer’s Disease (AD) pathology.

**Results:**

50.6% of the whole sample, including 28.7% cognitively normal and 66.7% of patients with mild cognitive symptoms, had ≥1 NPI symptoms. Frequencies of symptom clusters were largely similar between stroke subtypes. Compared to patients with cardioembolic stroke and intracranial haemorrhage, those with TIA had less frequent mood disturbance. Stroke severity at admission and MTLA were the most robust correlates of symptoms. FLA was associated with behavioral problems cluster only. Frequency of symptom clusters did not differ between patients with and without significant amyloid retention.

**Conclusion:**

Frequency of neuropsychiatric symptoms increased with level of cognitive impairment but was largely similar between stroke subtypes. Stroke severity and MTLA were associated with neuropsychiatric symptoms. AD pathology appeared to be unrelated to neuropsychiatric manifestations but further studies with larger sample size are required to substantiate this finding.

## Introduction

Neuropsychiatric symptoms are associated with a wide range of brain disorders including stroke and are strong predictors of adverse outcomes.[1, 2] Neuropsychiatric symptoms generally follow cognitive impairment, which is a prevalent complication of stroke.[3]. Although studies had examined neuropsychiatric disturbance in patients with stroke,[4] there is relatively little data on the manifestations of neuropsychiatric symptoms in patients with different levels of cognitive functioning and stroke subtypes.[5, 6] Moreover, most studies examined the relationship between location of stroke lesions with neuropsychiatric symptoms,[7–12] the impacts of chronic brain changes (e.g. chronic ischemic lesions and atrophy) and concomitant Alzheimer’s Disease (AD) pathology upon neuropsychiatric manifestations in patients with stroke or transient ischemic attack (TIA) are less clear. Utilizing data from a stroke cognitive registry,[13] the objectives of this study are 1) to examine the frequencies of neuropsychiatric symptoms and symptom clusters by cognitive level and stroke subtype in patients recently admitted for stroke or TIA, and 2) to evaluate the effects of demographic, clinical and cognitive factors as well as chronic brain changes upon neuropsychiatric symptom clusters. Furthermore, we performed *in vivo* amyloid imaging in a subset of sample to investigate the contribution of Alzheimer’s disease (AD) pathology to the neuropsychiatric manifestations in these patients.

## Materials and Methods

This study was approved by the Joint Chinese University of Hong Kong–New Territories East Cluster Clinical Research Ethics committee and written informedconsent was obtained from each participant.

### Subjects

This is a hospital-based, cross-sectional observational study. Participants consisted of a sample of consecutive patients admitted to the acute stroke unit of a university-affiliated hospital in the New Territories East region in Hong Kong between January 2009 and December 2010 due to stroke or TIA recruited in the on-going STRIDE (Stroke Registry Investigating Cognitive Decline) study, which aims to investigate the course and mechanisms of cognitive decline in patients with stroke or TIA.[13] An ischemic stroke was defined as clinical evidence of cerebral injury based on symptoms lasting more than 24 hours with other etiologies excluded. A TIA was identified as the presence of transient neurological deficits lasting fewer than 24 hours with absence of infarcts/haemorrhage evident on neuroimaging. Patients who were able to participate in cognitive assessment were eligible to participate in the STRIDE study. Additional informed consent from a proxy was obtained for patients who were mentally incompetent to give informed consent (e.g. dementia). Exclusion criteria included known history of dementia before the index stroke (i.e. prestroke dementia), significant sensorimotor and language impairment precluding participation in cognitive testing. Informants were invited to provide information regarding the cognitive, emotional, behavioral and functional aspects of the patients. Among the 1,013 patients recruited into the STRIDE study, 518 (51%) informants completed assessment in this substudy.

### Medical History

Information on demographic and vascular risk factors collected during the acute admission period (S1 Table). Definitions for the vascular risks are described previously.[13] Based on clinical presentation and neuroimaging data, patients were categorized into TIA and the following stroke subtypes based on the Trial of Org 10172 in Acute Stroke Treatment (TOAST) classification:[14] large artery atherosclerosis, small-artery occlusion, cardioembolism (CE), intracranial haemorrhage (ICH) and TIA. Score on the National Institute of Health Stroke Scale (NIHSS) obtained upon the index admission was recorded.

### Structural Neuroimaging

As part of the standard stroke service, non-contrast brain computed tomography (CT) was performed with a multidetector row clinical CT scanner for all patients upon arrival to the emergency room. For patients whose stroke subtypes could not be classified based on CT and clinical parameters, brain magnetic resonance imaging (MRI) was performed within one week from admission. MRI was performed on a 1.5-Tesla scanner (Sonata, Siemens Medical, Erlangen, Germany) or a 3.0-T scanner (Achieva 3.0 T TX Series, Philips Medical System, Best, the Netherlands) with the following sequences obtained: diffusion weighted imaging (DWI), axial gradient echo (GE), axial spin echo (SE) T1-weighted fast field echo (FFE), turbo spin echo (TSE) proton density (PD) and T2–weighted, axial FLAIR (Fluid Attenuated Inversion Recovery), and Time-of-Flight (TOF) MRA for 1.5-T MRI; and DWI, blood sensitive venous bold sequence, axial SE T1-weighted FFE, TSE T2–weighted, axial FLAIR and TOF MRA for 3-T MRI. Among the 518 patients included in this study, 264 (51%) had MRI. The following neuroimaging markers of chronic brain changes were obtained: white matter changes (WMC), the presence of old infarcts, ventricular-brain ratio (VBR) as a measure of global or subcortical atrophy, frontal lobe atrophy (FLA) and medial temporal lobe atrophy (MTLA). WMC was rated on axial FLAIR or CT scan using the Age-Related White Matter Changes (ARWMC) scale.[15] VBR was graded on axial MRI or CT and global or subcortical atrophy was defined as 4^th^ quartile of VBR.[16] Frontal lobe atrophy was defined as a rating of ≥1 on a 3-point (0–2) scale performed on axial T1-weighted MRI or CT [17], and MTLA as ≥2 on the Schelten’s scale rated on coronal images on T1-weighted MRI or CT.[18] Ratings were performed on MRI among patients with both CT and MRI. Intraclass correlation coefficient for interrater agreement for WMC, VBR and MTLA were 0.85–0.99 for CT and 0.75–0.93 for MRI.[13]

### Amyloid Imaging

Twenty-four patients underwent ^11^C-Pittsburg Compound B Positron Emission Tomography (^11^C-PiB PET). Details are described previously.[13, 19] The global standardized uptake value (SUV) of PIB retention at 35 min after injection was used as the index of amyloid load. Subjects with global SUV >1.5 were considered as having AD-like PIB binding.[20] ^11^C-PiB PET was performed by a specialist in nuclear medicine (E.L.).

### Assessment of Neuropsychiatric Symptoms

Study assessment was performed by trained research assistants and took place between 3 and 6 months post index event. The validated Chinese version of the 12-item Neuropsychiatric Inventory (NPI)[21] was administered to the informants during the study visit. It covers 12 neuropsychiatric symptoms including delusions, hallucinations, agitation/aggression, dysphoria/depression, anxiety, euphoria/elation, apathy/indifference, disinhibition, irritability/lability, aberrant motor behaviors, nighttime disturbances and appetite/eating disturbances of patients. Individual symptom score (0–12 point) is calculated by multiplying the frequency (0–4 point) and severity (0–3 point) scores. To provide a clinically meaningful interpretation of the symptoms, individual symptoms were further grouped into four symptom clusters according to a factor structure derived from a previous confirmatory factor analysis (CFA) conducted in local patients with AD.[22] The four symptom clusters included 1) *Behavioral Problems* (agitation/aggressiveness, disinhibition, irritability, and aberrant motor behavior); 2) *Psychosis* (delusions and hallucinations); 3) *Mood disturbance* (depression, anxiety, sleep, appetite, and apathy); and 4) *Euphoria* as a standalone factor. In view of the possible differences in neuropsychiatric manifestations between AD and patients with stroke or TIA, CFA was repeated in the entire study sample, which showed an identical four-factor structure (Table 1). Frequency of symptom clusters was determined as the number of patients with score of >0 in the respective symptom cluster.

**Table 1:** Standardized factor loadings for the four-factor model derived in confirmatory factor analysis.

NPI Item	Behavioral Problems	Psychosis	Mood Disturbance	Euphoria
8. Disinhibition	0.785***			
3. Agitation/Aggression	0.756***			
9. Irritability/Lability	0.695***			
10. Aberrant motor behaviors	0.685***			
2. Hallucinations		0.810***		
1. Delusions		0.785***		
4. Depression			0.743***	
5. Anxiety			0.707***	
11. Sleep and night time disturbance			0.546***	
7. Apathy/Indifference			0.638***	
12. Appetite/Eating disturbance			0.484***	
6. Elation/Euphoria				1.000

***p <0.001

CFA performed using weighted least squares with mean and variance adjustment (WLSMV)

Goodness-of-fit statistics of the four factor model: Chi-squared statistic = 50.47, df = 49, p = 0.415; Comparative Fit Index = 0.998; Tucker–Lewis Index = 0.997; Root Mean Square Error of Approximation = 0.008; Weighted Root Mean Square Residual = 0.737

### Assessment of Cognitive Functions

Cognitive functions were evaluated using the Cantonese version of the Mini-Mental State Examination (MMSE).[23] Based on all available clinical and psychometric information, the Clinical Dementia Rating Scale (CDR)[24] was administered to categorize patient’s current cognitive ability into five levels: 0 = no cognitive impairment; 0.5 = Mild Cognitive Impairment; 1 = Mild Dementia; 2 = Moderate Dementia and; 3 = Severe Dementia. Based on the CDR ratings, patients were grouped into three cognitive levels: Normal (CDR 0), Mild Cognitive Symptoms (CDR 0.5) and Dementia (CDR 1–3).

### Statistical Analysis

To evaluate potential selective biases, comparisons of demographic, cognitive, stroke severity and stroke subtype and neuroimaging features were made between patients recruited into this substudy and those recruited in the STRIDE study but not this substudy. Continuous variables were first checked for normality and were compared using Mann-Whitney *U* test or independent sample *t* test as appropriate. For categorical variables, Chi-squared test or Fisher’s Exact Test was used. Analysis of covariance adjusting for age, gender and education were used for group comparison of MMSE score to control for potential confounding effects. Because scores on the individual NPI symptoms and clusters were severely skewed, NPI responses were dichotomized into absence or presence in the analysis.[25] Logistic regression analysis was used to identify factors associated with presence of symptom clusters. Candidate independent variables including age, sex, year of education, presence of hypertension, diabetes mellitus, hyperlipidemia, atrial fibrillation, ischemic heart disease or congestive heart failure, any heart disease, stroke subtypes, prior stroke or TIA, MMSE, NIHSS at admission, ARWMC total score, presence of old infarcts, VBR, FLA and MTLA, were first submitted to individual univariable regression models and further entered into multivariable regression models if *p* value was ≤0.1 in the respective univariable model. Logistic regression analysis was not performed for Euphoria because of the extremely small number of patients (*n* = 7) reported having this symptom. For analysis on ^11^C-PiB PET data, age, sex, education level and frequency of NPI symptom cluster were compared between PiB-ve and PiB+ve groups using Mann-Whitney U test or Fisher’s exact test as appropriate. Statistical significance was determined as *p*<0.05 in the regression models. IBM SPSS Statistics version 21 and M-Plus version 6.1 were used for statistical analysis.

## Results

Five hundred and eighteen patients participated in this study. The mean age of the sample was 71.1 (*SD* = 11.0) years; 244 (51%) were female. Two hundred and fifty one (48.5%), 204 (39.4%) and 63 (12.1%) patients were rated as having normal cognition, mild cognitive symptoms and dementia, respectively. The number (%) for stroke subtypes and TIA were as follow: LAD 139 (26.8%), SAO 138 (26.7%), CE 73 (14.1%), ICH 40 (7.7%), Others 55 (10.6%) and TIA 73 (14.1%). A detailed summary of demographic, clinical, cognitive and neuroimaging data by cognitive level and stroke subtype are shown in the S1 Table. Missing data was below 5% for all variables collected. Compared to the STRIDE subjects who did not participate in this study, participants in this study were significantly older, less educated, had a higher proportion of female, had more severe strokes and poorer cognitive functions. Included patients also had a higher frequency of FLA, MTLA, old infarcts, and higher ARWMC score and VBR. There were no differences in terms of the proportion of stroke subtypes.

### Frequency of Symptom Clusters by Cognitive Level and Stroke Subtype/TIA

**Fig 1:**
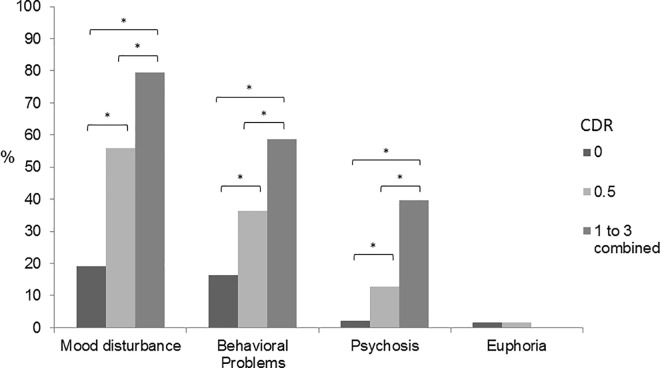
Frequency of neuropsychiatric symptom clusters across cognitive levels. * *p*<0.01

**Fig 2:**
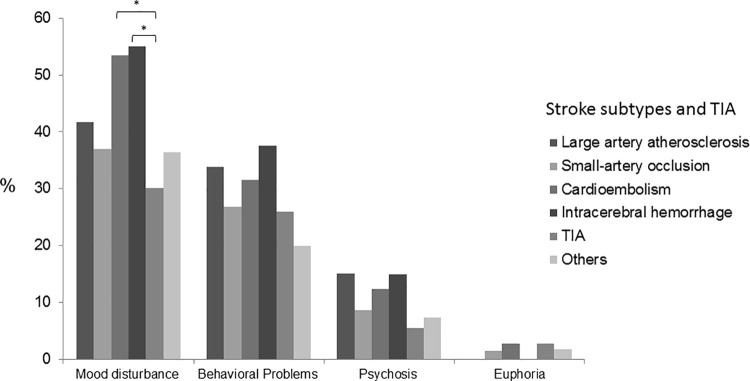
Frequency of neuropsychiatric symptom clusters stroke subtypes and TIA. * *p*<0.01

Study assessment took place at a mean of 158.5 (*SD* = 35.2) days after the index admission. Overall, 262 (50.6%) among the 518 patients reported ≥1 symptom on the NPI. Fig 1 shows the frequencies of symptom cluster across the three cognitive levels. Frequency increased with severity of cognitive impairment for all symptom clusters except euphoria (*p* = 0.68). 28.7% of the Cognitively Normal group, 66.7% of the Mild Cognitive Symptoms group and 85.7% of the Dementia group had ≥1 symptoms. Among all cognitive levels, the most frequent symptom cluster was mood disturbance, followed by behavioral problems, psychosis and euphoria. Euphoria was rare, occurring in only 4 and 3 patients in the normal and mild cognitive symptoms group, respectively. Fig 2 shows the symptom clusters frequencies across stroke subtypes. Across the stroke subtypes, mood disturbance was the most frequent, followed by behavioral problems, psychosis and euphoria. Overall symptom frequency did not differ between stroke subtypes (*p*>0.05). Pairwise comparisons between stroke subtypes and TIA revealed a significantly lower frequency of mood disturbance in TIA compared to CE and ICH groups (*p*<0.01).

### Correlates of Neuropsychiatric Symptom Clusters

Table 2 shows the results of multivariable logistic regression models for the any symptom and symptom clusters except euphoria. Among the clinical variables, NIHSS was consistently associated with the presence of any symptoms and all symptom clusters in the multivariate models. MMSE was generally associated with any symptoms and all symptom clusters in the univariable models (data not shown), but its effects upon behavioral problems and mood disturbance clusters disappeared after adjustment of confounding factors in the multivariable models. Among the neuroimaging variables, MTLA consistently accounted for the presence of any symptoms and all symptom clusters in the multivariable models even after adjusting for the clinical variables including MMSE and NIHSS. FLA was associated with the behavioral disturbance cluster only. WMC, VBR, and presence of old infarcts were not associated with the presence of any symptom or symptom cluster in the multivariate models.

**Table 2:** Predictors for presence of NPI symptom clusters in multivariable logistic regression models.

	Any symptoms		Behavioral Problems		Psychosis		Mood Disturbance	
	OR	95% CI	OR	95% CI	OR	95% CI	OR	95% CI
Age	0.97	0.95–0.99	NS		NS		NS	
Any Heart Disease	1.96	1.09–3.52	NS		NS		NS	
Prior Stroke or TIA	NS		NS		1.77	1.07–2.93	NS	
MMSE	0.92	0.88–0.96	NS		0.96	0.92–0.99	NS	
NIHSS at admission	1.09	1.04–1.14	1.06	1.02–1.10	1.06	1.02–1.10	1.07	1.02–1.11
FLA	NS		1.62	1.06–2.47	NS		NS	
MTLA	1.99	1.25–3.18	1.61	1.01–2.57	2.13	1.35–3.37	1.71	1.07–2.74

OR = Odds Ratio; CI = Confidence Interval; NS = not significant

Table showing independent variables significant in one or more symptom clusters only. Candidate variables examined in univariate regression included age, sex, year of education, hypertension, diabetes mellitus, hyperlipidemia, any heart disease, smoking, drinking, individual stroke subtypes (coded as dummy variables), prior stroke/TIA, MMSE, NIHSS at admission, ARWMC Total score, presence of old infarcts, VBR 4th quartile, FLA and MTLA

Regression analysis not performed for Euphoria as it was reported only in seven patients

### Comparison of frequency of symptom clusters between patients with and without significant amyloid uptake

We examined the contribution of AD to manifestation of neuropsychiatric manifestation. A total of 24 patients underwent ^11^C-PiB PET examination. Those who had ^11^C-PiB PET examination were older, less educated, had poorer cognitive functions as well as more frequent ratings in all symptom clusters (except euphoria) than those without (*p*<0.01). There was no group difference in the frequency of NPI symptom clusters between patients with and without significant amyloid uptake suggestive of AD (Table 3).

**Table 3:** Comparisons between patients with and without significant amyloid retention on ^11^C-PiB PET.

	Amyloid-ve	Amyloid+ve	p
n	16	8	
Age in years	77.1 (6.3)	78.4 (4.2)	0.603
Female (n [%])	5 (62.5%)	7 (43.8%)	0.667
Education in years	3.6 (3.4)	1.4 (1.9)	0.108
MMSE	15.5 (7.8)	13.5 (10.0)	0.569
Presence of NPI Symptom Clusters (n [%])			
Behavioral Problems	10 (62.5%)	5 (62.5%)	0.999
Psychosis	5 (31.3%)	3 (37.5%)	0.999
Mood disturbance	11 (68.8%)	6 (75.0%)	0.999
Euphoria	1 (6.3%)	0 (0.0%)	0.999

Age, education and MMSE are shown in median (interquartile range)

## Discussion

In this study we showed that half of patients, including approximately one-third of patients with normal cognition and two-thirds of those with mild cognitive symptoms, had one or more neuropsychiatric symptoms 3 to 6 months after stroke or TIA. Symptom frequency was generally similar between stroke subtypes, with the exception that patients with TIA had less frequent mood disturbance compared to those with CE stroke and ICH. NIHSS and MTLA were the most robust correlates of symptom clusters. Based on the data from a small subset of patients (n = 24), there appeared to be no evidence of an association between concomitant AD with the neuropsychiatric manifestations in these patients.

Neuropsychiatric manifestations in patients with stroke or TIA having no or mild cognitive symptoms is not clear. Using the NPI, a study (*n* = 60) showed that 95% of patients with mild to severe cognitive symptoms poststroke had one or more neuropsychiatric symptoms three months poststroke.[5] Another study (*n* = 41) showed a frequency of 85% in patients with mild cognitive impairment of vascular etiology.[6] In contrast, with a substantially larger sample (*n* = 518), we found an overall frequency of 50.6% in the whole sample and a lower frequency of 66.7% in patients with mild cognitive symptoms. The latter figure appeared to be higher than that reported in patients with mild cognitive impairment in the general population (43–51%).[25–27] Overall symptom frequency (28.7%) in our cognitively normal group was similar to, or slightly higher than, 16–25% reported in the normal elderly persons.[25, 27] Regarding TIA and stroke subtypes, TIA patients had a lower frequency of mood disturbance compared to those with CE and ICH, but otherwise frequencies were similar. Note that the frequency of 42.5% in TIA patients appeared to be higher than that reported in the normal elderly population.[25–27] This elevated frequency may represent subtle but lasting neurobiological changes beyond the resolution of physical symptoms [28] or persistent perfusion deficits after TIA.[29] We also found that euphoria was rare (1.3%) among this population. Note that poststroke emotional incontinence, which was found in 7.6% in a similar local sample in a previous study, might manifest as pathological laughter and interpreted as signs of euphoria by the informants on the NPI.[30]

With regard to factors associated with neuropsychiatric manifestations, we found that stroke severity at admission had the most robust associations with the overall symptoms and symptom clusters.[31] In terms of the effects of chronic brain changes, we demonstrated a robust effect of MTLA, which is an early marker of degenerative neuropathology including AD.[18, 32] However, the effect of MTLA might be independent of cognitive impairment as the association remained after MMSE was adjusted in the regression model. This finding suggested that MTLA might contribute to the development of neuropsychiatric symptoms via neural pathways that are more or less independent from those involved in cognitive functioning. For example, the medial temporal lobe has extensive connections with the frontal lobes and limbic system and thus its cell loss may disrupt pathways involved in emotional and behavioral regulations.[33, 34] In addition to MTLA, we showed that FLA was specific to behavioral disturbance. The relation between the frontal lobes and socio-emotional disturbance and behavioral disorders are well established.[33] On the other hand, we did not find any effects from WMC, global or subcortical atrophy and old infarcts. Although the “vascular depression” hypothesis [35] was partly supported in the general elderly population, [36] our findings were in agreement with clinical studies in stroke showing no effect of WMC upon depressive symptoms.[37, 38] However, low WMC severity in the sample might explain this lack of association. It is also possible that after a stroke or TIA, more salient neurobiological changes might override the effects of chronic vascular diseases.

However, despite the robust effect of MTLA, which is often considered a marker of AD, [32][18] analysis of the ^11^C-PiB PET data showed no evidence that significant amyloid retention contributed to the neuropsychiatric manifestations at 3 to 6 months after stroke or TIA. In our sample, the frequencies of symptom clusters were almost identical between those with and without amyloid retention. Our findings are in contrast to that reported in a previous study conducted on patients with subcortical vascular cognitive impairment that amyloid retention was related to an increased likelihood of delusions and irritability.[39]

There are a number of limitations in our study. First, we used NPI but not formal clinical diagnosis as the outcome measures. Despite its widespread use, the NPI is a brief instrument with known limitations [40]. Moreover, despite our relatively large overall sample size, further grouping by cognitive level and stroke subtype resulted in small and uneven group sizes. Also, selective biases were evident as patients recruited in this study were older, less educated and more impaired than the other STRIDE participants. Likewise, patients with prestroke dementia or very severe dementia poststroke precluding cognitive assessment were not included in the STRIDE study. Furthermore, as cognitive status was defined clinically by ratings on the CDR, the possibility of prodromal MCI or dementia, which might have exerted an influence upon the neuropsychiatric manifestations, could not be completely excluded given that 27.1% of patients rated as cognitively normal had MTLA. Also, we only examined a limited set of chronic brain changes and did not include measures of acute infarct as only half of the patients had DWI, thus we could not evaluate the interactions between acute and chronic brain changes upon neuropsychiatric manifestations. Moreover, reliability of rating of chronic brain lesions was compromised as such ratings were performed using CT and MRI of different field strengths. Likewise, small and infratentorial infarcts and mild WML might not have been visualized well on CT. Furthermore, we did not obtain measures of the cerebellum or brainstem lesions known to correlate with pathological crying or laughter that might be interpreted as signs of depression or euphoria by the informants.[41] Last but not least, only a small subset of patients underwent ^11^C-PiB PET examination and these patients were older, less educated and more cognitively impaired than the entire sample. Future studies are needed to validate our findings.

## Supporting Information

S1 Dataset The minimal dataset contains all the data pertinent to the analysis and results reported in this manuscript. (XLSX)

S1 Table Summary of demographic, clinical and neuroimaging features by cognitive level and stroke subtype (DOCX)
