# Supplementary material for: Neuropsychiatric Symptom Clusters in Stroke and Transient Ischemic Attack by Cognitive Status and Stroke Subtype: Frequency and Relationships with Vascular Lesions, Brain Atrophy and Amyloid
Source: PLoS One. 2016 Sep 15;11(9):e0162846. doi: 10.1371/journal.pone.0162846 (PMC5025073; doi:10.1371/journal.pone.0162846)
Supplement: S1 Table — (DOCX) [file pone.0162846.s002.docx]

| S1 Table. Summary of demographic, clinical and neuroimaging features by cognitive level and stroke subtype | | | | | | | |  |  |
| --- | --- | --- | --- | --- | --- | --- | --- | --- | --- |
|  | Cognitive Level | | | Stroke Subtype | | | | | |
|  | Normal | Mild Cognitive Symptoms | Dementia | LAD | SAO | CE | ICH | TIA | Others |
| N | 251 | 204 | 63 | 139 | 138 | 73 | 40 | 73 | 55 |
| Age in years | 68.7 (10.7) | 72.2 (10.7) | 79.9 (8.4) | 73.0 (10.1) | 69.7 (11.5) | 74.9 (9.1) | 71.0 (13.3) | 69.1 (10.8) | 70.8 (11.6) |
| Female (n [%]) | 117 (46.6%) | 111 (54.4%) | 36 (57.1%) | 62 (44.6%) | 75 (54.3%) | 42 (57.5%) | 14 (35.0%) | 44 (60.3%) | 27 (49.1%) |
| Education in years | 6.0 (4.6) | 4.2 (4.3) | 3.2 (3.6) | 5.0 (4.4) | 5.5 (4.5) | 4.5 (4.7) | 4.9 (4.5) | 4.4 (4.4) | 4.7 (4.3) |
| *Vascular Risk Factors and Clinical Data* | |  |  |  |  |  |  |  |  |
| Hypertension | 168 (66.9%) | 135 (66.2%) | 50 (79.4%) | 99 (71.2%) | 92 (66.7%) | 48 (65.8%) | 29 (72.5%) | 51 (69.9%) | 34 (61.8%) |
| Hyperlipidemia | 152 (60.6%) | 115 (56.4%) | 27 (42.9%) | 97 (69.8%) | 87 (63.0%) | 33 (45.2%) | 6 (15.0%) | 43 (58.9%) | 28 (50.9%) |
| Diabetes mellitus | 80 (31.9%) | 66 (32.4%) | 30 (47.6%) | 49 (35.3%) | 48 (34.8%) | 27 (37.0%) | 11 (27.5%) | 22 (30.1%) | 19 (34.5%) |
| Smoking | 35 (13.9%) | 24 (11.8%) | 4 (6.3%) | 23 (16.5%) | 20 (14.5%) | 3 (4.1%) | 2 (5.0%) | 7 (9.6%) | 8 (14.5%) |
| Drinking | 13 (5.2%) | 9 (4.4%) | 0 (0.0%) | 6 (4.3%) | 5 (3.6%) | 1 (1.4%) | 3 (7.5%) | 5 (6.8%) | 2 (3.6%) |
| Prior Stroke or TIA | 35 (13.9%) | 86 (42.2%) | 15 (23.8%) | 21 (15.1%) | 31 (22.5%) | 11 (15.1%) | 5 (12.5%) | 11 (15.1%) | 7 (12.7%) |
| Atrial fibrillation | 30 (12.0%) | 35 (17.2%) | 14 (22.2%) | 3 (2.2%) | 2 (1.4%) | 56 (76.7%) | 8 (20.0%) | 7 (9.6%) | 3 (5.5%) |
| Ischemic heart disease | 21 (8.4%) | 21 (10.3%) | 6 (9.5%) | 13 (9.4%) | 8 (5.8%) | 12 (16.4%) | 1 (2.5%) | 10 (13.7%) | 4 (7.3%) |
| Congestive heart failure | 4 (1.6%) | 4 (2.0%) | 7 (11.1%) | 4 (2.9%) | 1 (0.7%) | 6 (8.2%) | 0 (0.0%) | 2 (2.7%) | 2 (3.6%) |
| Any Heart Disease | 46 (18.3%) | 53 (26.0%) | 22 (34.9%) | 16 (11.5%) | 10 (7.2%) | 62 (84.9) | 8 (20.0%) | 18 (24.7%) | 7 (12.7%) |
| NIHSS^†^ | 3.0 (4.0) | 4.0 (5.0) | 6.0 (8.50) | 5.0 (5.0) | 3.0 (4.0) | 7.0 (8.0) | 9.0 (10.0) | 1.0 (2.0) | 4.0 (5.0) |
| MMSE | 26.2 (3.4) | 22.3 (5.7) | 12.9 (5.3) | 21.8 (6.7) | 24.6 (5.4) | 21.5 (6.8) | 20.5 (7.2) | 24.6 (5.1) | 23.9 (5.6) |
| *Neuroimaging Variables* | |  |  |  |  |  |  |  |  |
| ARWMC scale total score^†^ | 2.0 (6.0) | 2.0 (7.0) | 6.0 (8.0) | 4.9 (4.0) | 4.6 (4.6) | 2.9 (4.0) | 5.0 (4.4) | 2.3 (3.5) | 2.5 (3.3) |
| Old infarct Presence | 148 (59.0%) | 133 (65.2%) | 47 (74.6%) | 105 (75.5%) | 91 (65.9%) | 40 (54.8%) | 29 (72.5%) | 33 (45.2%) | 30 (54.5%) |
| VBR | 0.31 (0.04) | 0.32 (0.06) | 0.35 (0.05) | 0.32 (0.06) | 0.32 (0.05) | 0.33 (0.04) | 0.33 (0.05) | 0.31 (0.05) | 0.32 (0.04) |
| FLA Presence | 102 (40.6%) | 86 (42.2%) | 28 (44.4%) | 63 (45.3%) | 52 (37.7%) | 33 (45.2%) | 19 (47.5%) | 26 (35.6%) | 23 (41.8%) |
| MTLA Presence | 68 (27.1%) | 78 (38.2%) | 41 (65.1%) | 53 (38.1%) | 46 (33.3%) | 30 (41.1%) | 22 (55.0%) | 16 (21.9%) | 20 (36.4%) |
| ^†^Shown in median (interquartile range) | | | | | | | | | |
| Abbreviations: TIA=Transient Ischemic Attack; NIHSS=National Institute of Health Stroke Scale; MMSE=Mini-Mental State Examination; ARWMC=Age-Related White Matter Change Scale; VBR=Ventricular-Brain Ratio; FLA=Frontal Lobe Atrophy; MTLA=Medial Temporal Lobe Atrophy | | | | | | | | | |
